# Enhanced Anticancer Activity of 7MeERT over Ertredin: A Comparative Study on Cancer Cell Proliferation and NDUFA12 Binding

**DOI:** 10.3390/biom14091197

**Published:** 2024-09-23

**Authors:** Sonoko Atsumi, Chisato Nosaka, Takefumi Onodera, Hayamitsu Adachi, Takumi Watanabe, Manabu Kawada, Masabumi Shibuya, Se In Park, Ho Jeong Kwon

**Affiliations:** 1 Institute of Microbial Chemistry, Shinagawa-ku, Tokyo 141-0021, Japan; nosaka@bikaken.or.jp (C.N.); onoderat@bikaken.or.jp (T.O.); adachih@bikaken.or.jp (H.A.); twatanabe@bikaken.or.jp (T.W.); kawadam@bikaken.or.jp (M.K.); 2 Gakubunkan Institute of Physiology and Medicine, Jobu University, Takasaki-shi, Isesaki 370-1393, Japan; shibuya@jobu.ac.jp; 3 Chemical Genomics Leader Research Laboratory, Department of Biotechnology, College of Life Science and Biotechnology, Yonsei University, Seoul 03722, Republic of Korea; tpdls0303@yonsei.ac.kr

**Keywords:** antiproliferative activity, Ertredin, glioblastoma, NDUFA12, oxidative phosphorylation, tumorigenesis, 7MeERT

## Abstract

We have previously identified Ertredin (3-(2-amino-5-bromophenyl) quinoxalin-2(1H)-one) as a compound that suppresses 3D spheroid formation and tumorigenesis in NIH3T3 cells induced by *Epidermal Growth Factor Receptor* variant III (*EGFRvIII*) transduction. One of its targets has been shown to be NDUFA12 (NADH Dehydrogenase (Ubiquinone) 1 Alpha Subcomplex Subunit 12), a component protein of oxidative phosphorylation complex I. In this report, we compared the growth inhibitory activity of Ertredin with its methylated analogue 7MeERT (3-(2-amino-5-bromophenyl)-7-methylquinoxalin-2(1H)-one) on human cancer cells. 7MeERT induced the inhibition of the proliferation of various cancer cells similarly to Ertredin and showed higher activity in glioblastoma cells, A431 cells overexpressing *EGFR* (wild type), and multiple myeloma cells. Molecular docking analysis and a Cellular Thermal Shift Assay (CETSA) suggested that 7MeERT binds to NDUFA12 similarly to Ertredin. The binding of 7MeERT and Ertredin to NDUFA12 in glioblastoma was further supported by the inhibition of the oxygen consumption rate. These results suggest that 7MeERT also binds to NDUFA12, inhibits oxidative phosphorylation, and has a higher anti-cancer cell growth inhibitory activity than Ertredin.

## 1. Introduction

EGFRvIII is a mutant form of EGFR that is constitutively activated due to the deletion of the ligand binding domain [1,2,3,4,5]. It is expressed in more than 40% of patients with unresectable glioblastoma and is associated with the malignancy of the cancer [6]. Mouse NIH3T3 cells (NIH3T3/EGFRvIII) that overexpress the EGFRvIII gene acquire the ability to form 3D spheroids on low-adhesion plates and develop tumorigenic capabilities in vivo [7].

In our previous studies, we identified Ertredin as a compound that inhibits 3D spheroid formation in NIH3T3/EGFRvIII cells and also suppresses tumor formation in animal models [7]. Our research further identified NDUFA12, a subunit of mitochondrial respiratory chain complex I, as the target molecule of Ertredin [8]. Complex I, also known as NADH oxidoreductase, is the largest enzyme of the oxidative phosphorylation system, consisting of 45 protein subunits [9,10]. Among these subunits, NDUFA12 plays an essential role as an accessory protein, contributing to the stability and assembly of complex I [11,12]. Mutations in NDUFA12 have been associated with mitochondrial disorders such as Leigh syndrome, highlighting its significance in mitochondrial function [13].

Recent studies have demonstrated that targeting mitochondrial metabolism, particularly complex I, offers a promising approach for cancer therapy. Inhibitors of complex I can disrupt the energy production in cancer cells, which heavily rely on oxidative phosphorylation for survival and growth [14,15,16]. Given that NDUFA12 is involved in the electron transfer process and is essential for energy metabolism, it has become a potential therapeutic target in cancers that rely on mitochondrial respiration.

By binding to NDUFA12, Ertredin may affect the energy metabolism of cancer cells, thereby contributing to its antiproliferative activity. Indeed, in NIH3T3/EGFRvIII cells, Ertredin showed significant inhibitory effects on respiratory chain complex I [7]. In this report, we conducted a comparative study of the antiproliferative activity of Ertredin and its methylated analogue 7MeERT against various human cancers and analyzed their mechanisms of action.

## 2. Materials and Methods

### 2.1. Chemicals and Antibodies

Ertredin was synthesized according to the previously reported method [7]. Detailed spectral data (NMR, IR, MS) for Ertredin have been previously published in [17], and are included in the supplementary information of [7]. 7MeERT, 3-phenylquinoxalin-2(1H)-one (PQXO), and quinoxalin-2(1H)-one (QXO) were purchased from Enamine (Kiev, Ukraine). Rabbit anti-NDUFA12 antibody was purchased from Novus (Centennial, CO, USA), and mouse anti-actin antibody was purchased from Abcam (Cambridge, United Kingdom). AG1478 and metformin were purchased from Fujifilm (Tokyo, Japan).

### 2.2. Cell Lines

NIH3T3 (mouse-derived fibroblasts), U87MG (human-derived glioblastoma), A431 (human-derived epidermoid carcinoma), HeLa (human-derived cervical cancer), and RPMI8226 (human-derived multiple myeloma) cells were purchased from the American Type Culture Collection. U251 (human-derived glioblastoma) and KMS-11 (human-derived multiple myeloma) cells were obtained from the Japanese Collection of Research Bioresources. NIH3T3/EGFRvIII cells were generated by introducing the EGFRvIII gene into NIH3T3 [7]. U251/EGFRvIII and U87MG/EGFRvIII cells were generated with the CSII-CMV-MCS-IRES2-Bsd vector, kindly provided by H. Miyoshi (RIKEN Tsukuba Institute). PCR was used to amplify EGFRvIII from the expression vector to construct NIH3T3/EGFRvIII, which was cloned into CSII-CMV-MCS-IRES2-Bsd at the NheI and XhoI sites. Adherent cells were cultured in Dulbecco’s Modified Eagle Medium with 10% FBS under both 2D and 3D conditions, and suspension cells were cultured in Roswell Park Memorial Institute 1640 medium containing 10% FBS. All cultures were maintained at 37 °C in a 5% CO_2_ incubator, with medium changes or passaging performed every 3 to 7 days.

### 2.3. Cell Proliferation Inhibition Assay

Cells (2 × 10^3^ to 7 × 10^3^ cells/100 μL per well) were seeded in 96-well culture plates. After the addition of the compounds, the cells were incubated for 3 days. This method describes the 2D cell culture procedure. For the 3D culture method, please refer to [7]. Control wells included cells treated with DMSO (vehicle control) and untreated cells (negative control) to account for the baseline cell growth. Additionally, known inhibitors of cell proliferation, such as AG1478, were used as positive controls. Subsequently, the MTT assay was performed by adding 3-(4,5-dimethylthiazol-2-yl)-2,5-diphenyltetrazolium bromide to a final concentration of 0.5 mg/mL. The plates were returned to the 5% CO_2_ incubator, and SDS was added after 3.5 h. Absorbance at 570 nm in each well was measured after 24 h using an ARVO reader (PerkinElmer).

### 2.4. Statistical Analysis

All experiments were conducted in triplicate, and the results are presented as the means ± standard deviations (SDs). IC_50_ values were determined using a nonlinear regression analysis. Specifically, the cell proliferation inhibition data were plotted, and the X-axis value corresponding to 50% inhibition on the Y-axis was manually read to calculate the IC_50_. All data analysis was performed using GraphPad Prism (version 9).

### 2.5. In Silico Docking Analysis

To analyze the interaction between 7MeERT and NDUFA12, a molecular docking analysis was performed using Discovery Studio Client v18.1.0.17334 software (Accelrys, San Diego, CA, USA) adopting the CHARMM (Chemistry at Harvard Macromolecular Mechanics) force field. The 3D structure of human NDUFA12 (Alphafold: AF-O97725-F1) was obtained from the AlphaFold Protein Database. 7MeERT was docked to the binding site of the protein and 70 poses were generated. The most predictive binding modes were determined based on several scoring functions (Ligscore1_Dreiding, Ligscore2_Dreiding, PLP1, PLP2, Jain, PMF, and PMF04), and the binding energies were determined by calculating the binding energy of the most predictive binding mode. Dock Ligands (CDOCKER) uses a CHARMM-based molecular dynamics (MD) scheme to dock ligands into a receptor binding site. CDOCKER E: −23.3355 kcal/mol.

The docking analysis was performed using the receptor structure ‘AF-O97725-F1_prep’, and the prepared ligand ‘7MeERT(1): All’ was used as the input ligand. The binding site was defined by a sphere with center coordinates (6.04436, 6.91881, −7.55937) and a radius of 8.5 Å. The analysis was set to identify the top 10 binding poses, with a clustering radius of 0.1 Å. Ten random conformations were generated for the ligand. Molecular dynamics simulations were performed with 1000 steps at a target temperature of 1000 K, and electrostatic interactions were included in the calculations. A total of 10 orientations were refined, allowing up to 300 bad orientations and using a Van der Waals energy threshold of 300. Simulated annealing was enabled, with 2000 heating steps at a target temperature of 700 K, followed by 500 cooling steps at 300 K. The CHARMM force field was used, and full potential was applied only during the final minimization step with a gradient tolerance of 0.1. The partial charges of the ligand were calculated using the Momany–Rone method, and the lattice was extended to 8.0 Å.

### 2.6. Physicochemical Properties (ADME/T) Prediction

The HITS program “https://hyperlab.hits.ai/ (assessed on 12 July 2024)” was used to predict interactions between 7MeERT, Ertredin, and the NDUFA12 protein and their physicochemical properties, including ADME/T prediction (Protein Data Bank ID: O97725.pdb). Binding energies were calculated to evaluate the strength of interaction between the compounds and NDUFA12. 

### 2.7. CETSA Method

The CETSA method was performed as described previously [8]. Briefly, U251/EGFRvIII cells (2 mL, density 3 × 10^6^ cells/mL) were treated with vehicle (DMSO) or Ertredin analogues (dissolved in DMSO) to produce different final concentrations and incubated for 1 h at 37 °C in a humidified incubator with 5% CO_2_. The cells were washed with PBS (2 × 2 mL), transferred to PCR tubes, and heated using a thermal cycler (Gene AtlasG, Astec, Fukuoka, Japan) at temperatures ranging from 40 °C to 60 °C for 3 min, followed by cooling to 25 °C. The temperature range was selected based on previous results [8], where Ertredin was shown to stabilize NDUFA12 between 45 °C and 55 °C in HepG2 cells. This range was used to ensure consistency with prior experiments and to assess potential shifts in thermal stability induced by Ertredin and 7MeERT in U251/EGFRvIII cells. Each sample was then mixed with 1/3 volume of NuPAGE™ LDS Sample Buffer (4X) (Thermo Fisher Scientific) with 25 mM DTT and boiled for 10 min before performing Western blotting.

### 2.8. Western Blotting

Western blotting was performed as described in reference [7]. A 10–20% SDS-PAGE was used for protein separation. Visualization was conducted using ECL Prime Western Blotting Detection Reagent, and detection was carried out with an ImageQuant LAS 4000 Mini (GE Healthcare Life Sciences). Band intensities were quantified using Image J (ver. 1.54d).

### 2.9. Oxygen Consumption Rate (OCR) Measurement

Cells (8 × 10^4^ cells per well) were seeded in a Seahorse XFe24 plate and incubated overnight at 37 °C in a 5% CO_2_ incubator. The next morning, the medium was replaced with Seahorse XF Base Medium containing final concentrations of 5.56 mM D-glucose and 2 mM glutamine. The plate was incubated in a CO_2_-free incubator for 1 h and then set up in a Seahorse XFe 24 Analyzer (Agilent Technologies). The OCR was measured under various conditions using an XF Mito Stress Test Kit (Agilent Technologies).

## 3. Results

### 3.1. Enhanced Antiproliferative Activity of 7MeERT in Various Cancer Cell Lines

**Figure 1:**
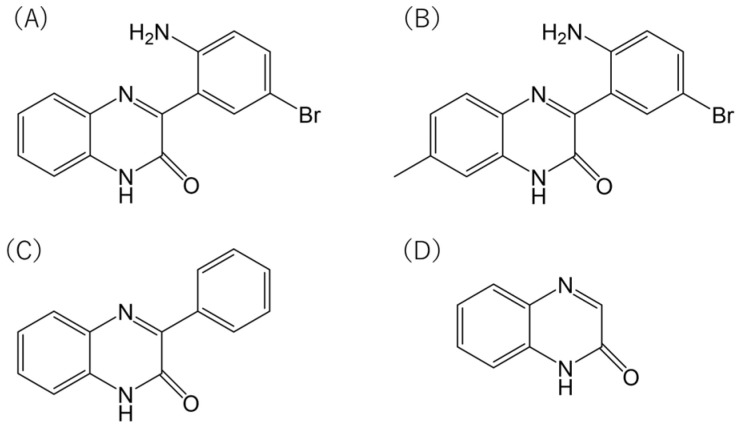
Structures of Ertredin analogues. (**A**) Ertredin [3-(2-amino-5-bromophenyl) quinoxalin-2(1*H*)-one]. (**B**) 7MeERT [3-(2-amino-5-bromophenyl)-7-methylquinoxalin-2(1*H*)-one]. (**C**) PQXO [3-phenylquinoxalin-2(1*H*)-one]. (**D**) QXO [quinoxalin-2(1*H*)-one].

**Figure 2:**
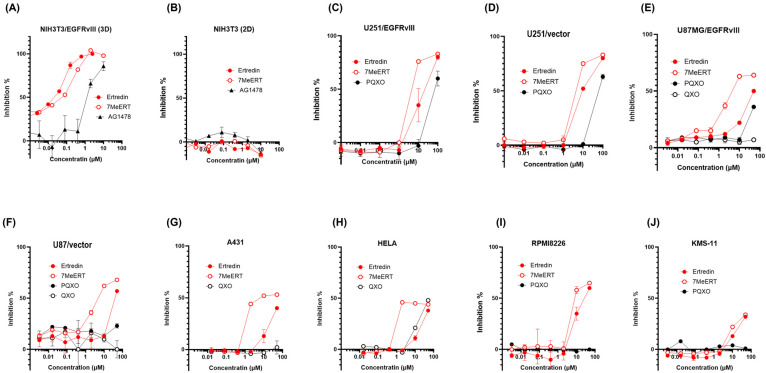
Growth inhibition curves of cells cultured for 3 days with Ertredin (red solid circle), 7MeERT (red open circle), AG1478 (triangle), PQXO (black solid circle), and QXO (black open circle) for (**A**) NIH3T3/EGFRvIII, (**B**) NIH3T3, (**C**) U251/EGFRvIII, (**D**) U251/vector, (**E**) U87MG/EGFRvIII, (**F**) U87MG/vector, (**G**) A431, (**H**) HeLa, (**I**) RPMI8226, and (**J**) KMS-11 cells.

The methylated analogue of Ertredin, 7MeERT (Figure 1B), exhibited antiproliferative activity in 3D cultures of mouse NIH3T3 cells expressing EGFRvIII (NIH3T3/EGFRvIII), second only to Ertredin (Figure 2A). The 3D antiproliferative activities of Ertredin and 7MeERT were significantly higher compared to the EGFR inhibitor AG1478 (Figure 2A). Conversely, as previously reported [7], Ertredin analogues did not show any antiproliferative activity in normal parental NIH3T3 cells (2D culture) (Figure 2B).

7MeERT inhibited the proliferation (2D) of human glioblastoma cells expressing EGFRvIII, U251/EGFRvIII (Figure 2C), and U87MG/EGFRvIII (Figure 2E) with an IC50 of 2 μM in both U251/EGFRvIII and U87MG/EGFRvIII cells, showing higher activity than Ertredin (IC50 = 10 μM in U251/EGFRvIII cells and IC50 = 20 μM in U87MG/EGFRvIII cells). Meanwhile, 7MeERT and Ertredin showed similar activities in control vector-introduced glioblastoma cells, suppressing growth regardless of EGFRvIII expression (Figure 2D,F, Supplementary Figure S1). 7MeERT also had higher antiproliferative activities in epidermoid carcinoma A431 cells overexpressing EGFR (IC50 = 5 μM, Figure 2G) and cervical cancer HeLa cells (IC50 = 1 μM, Figure 2H) compared to Ertredin (IC50 = 50 μM). Ertredin analogues also showed antiproliferative activity in RPMI8226 multiple myeloma cells (Figure 2I, Ertredin; IC50 = 16 μM, 7MeERT; IC50 = 8 μM), but only weak activity was detected in KMS-11 cells (Figure 2J, IC50 > 100 μM). The cell proliferation inhibition curves for various cancer cells were consistent between Ertredin and 7MeERT, suggesting a common inhibitory mechanism (Figure 2A–J). Moreover, 7MeERT showed higher antiproliferative activity against various cancer cells compared to Ertredin (Table 1). In addition, PQXO (Figure 1C) and QXO (Figure 1D) did not show significant activity in cells other than HeLa cells (Figure 2C–J and Table 1). This suggests that the Br and phenyl residues of Ertredin analogues are important for their antiproliferative activity against human cancer cells. The importance of these residues was consistent with the results observed in the structure–activity relationship study of Ertredin analogues for the inhibition of NIH3T3/EGFRvIII (3D culture) proliferation [7].

The results demonstrate that 7MeERT exhibits a higher antiproliferative activity than Ertredin in various cancer cell lines, including glioblastoma, epidermoid carcinoma, and cervical cancer. This suggests that methylation enhances the antiproliferative effect of Ertredin, making 7MeERT a more potent inhibitor of cancer cell proliferation.

### 3.2. Molecular Docking Analysis of 7MeERT Targeting NDUFA12

NDUFA12 has previously been identified as the target of Ertredin [8]. Therefore, we investigated whether 7MeERT also targets the same protein through a molecular docking analysis. To identify potential binding sites of 7MeERT within NDUFA12, we performed an in silico docking analysis using the 3D structure of NDUFA12 (AF-O97725-F1). We used CDOCKER, a CHARMm-based docking engine, for flexible ligand-based docking and refinement.

**Figure 3:**
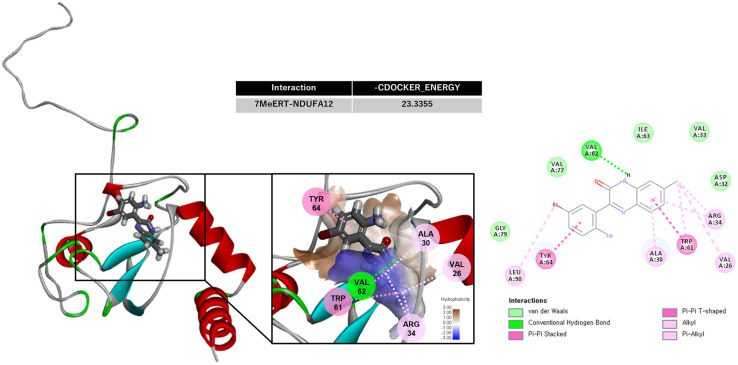
The in silico docking analysis. The 2D and 3D binding interactions of 7MeERT with the target NDUFA12 and potential binding sites of 7MeERT identified by the in silico molecular docking simulation.

CDOCKER is a docking algorithm available in molecular modeling software such as Discovery Studio Client v18.1.0.17334 (Accelrys, San Diego, CA, USA) that is commonly used to predict the binding modes and affinities of ligands to proteins. It calculates the energy of interaction when a ligand binds to the active site of the protein. The CDOCKER algorithm uses molecular dynamics to explore various poses (spatial orientations) of the ligand within the binding site, calculating the energy for each pose. This energy evaluation takes into account factors such as electrostatic interactions, hydrophobic interactions, and hydrogen bonding between the ligand and the protein. The ligand is scanned in multiple conformations (with different rotations and translations) with the goal of identifying the lowest energy pose that represents the most stable binding mode. A negative energy value indicates stable binding, and in general, the more negative the value, the stronger the interaction [18]. The calculated CDOCKER energy for the interaction between 7MeERT and NDUFA12 was −23.3355 kcal/mol (Figure 3), suggesting a stronger binding affinity of 7MeERT toward NDUFA12 compared to Ertredin, which had a CDOCKER energy of −17.2322 kcal/mol.

The in silico docking analysis detailed the interactions between 7MeERT and NDUFA12. In the 2D interaction diagram on the right, the specific parts of the 7MeERT compound that interact with the protein’s amino acids are as follows: The amide nitrogen (N-H) of 7MeERT forms a hydrogen bond with V62 (green dashed line). In this interaction, the nitrogen of the amide group acts as a hydrogen donor, while the carbonyl oxygen of V62 accepts the hydrogen. The quinoline ring structure (aromatic ring) forms a π-π stacking interaction with Y64 (pink dashed line), involving the parallel stacking of the aromatic rings of both the ligand and tyrosine. The quinoline ring structure also forms a π-π T-shaped interaction with W61 (pink dashed line), where the aromatic rings are arranged in a perpendicular (T-shaped) configuration. The aromatic ring and alkyl groups of 7MeERT participate in π–alkyl interactions with R34 (pink dashed line), where the electron cloud from the aromatic ring interacts with the alkyl groups of R34. In addition, A30 and V26 participate in π–alkyl interactions with the ligand (pink dashed lines), indicating interactions between the alkyl groups and the aromatic moieties of the ligand.

In summary, the amide nitrogen of 7MeERT forms a hydrogen bond with V62, while the quinoline ring participates in π-π stacking with Y64 and W61, and various alkyl and π–alkyl interactions occur with amino acids such as R34, A30, and V26. Notably, the presence of the methyl group in 7MeERT significantly enhances both π and alkyl interactions. Despite the relatively weak nature of alkyl interactions in biology and chemistry, their cumulative effect on chemical reactivity can be significant [19]. Thus, it can be inferred that these enhanced π and alkyl interactions contribute substantially to the increased binding energy observed between 7MeERT and NDUFA12 compared to Ertredin.

### 3.3. Comparative Analysis of 7MeERT and Ertredin with NDUFA12 Using Physical–Deep Learning Hybrid Virtual Screening

Using the Hyperlab program (HITS: hyperlab.hits.ai), we predicted the interaction between NDUFA12 and the drug candidates 7MeERT and Ertredin, as well as their physicochemical properties (ADME/T prediction) (Table 2). A lower hyper binding value indicates stronger binding to the target protein, corresponding to higher activity. This calculation resulted in binding scores of −3.7 kcal/mol (7MeERT-NDUFA12) and −3.3 kcal/mol (Ertredin–NDUFA12). The binding score represents the binding energy predicted by Hyperlab’s AI model, where a more negative value indicates greater binding stability. This score approximates the pIC_50_ divided by −1.34.

The analysis showed that the predicted binding energy with NDUFA12 for 7MeERT (−3.7 kcal/mol) was lower than for Ertredin (−3.3 kcal/mol), indicating a greater binding stability. Both drug candidates exhibited a TPSA (Topological Polar Surface Area) of 72 Å^2^, indicating good cell membrane permeability, and adhered to Verber’s (GSK) rule, suggesting a favorable ADME/T profile (TPSA ≤ 140 Å^2^, rotatable bonds ≤ 10). The predicted logP values, indicating lipid affinity, were 3.2 for 7MeERT and 2.9 for Ertredin, which were within the generally optimal range of 1 < logP < 3 for drug absorption, distribution, metabolism, and excretion, although this range may vary based on the target tissue, administration method, and mechanism of action. Both candidates were predicted to be metabolically stable (not degrading over 50% within 30 min at an initial concentration of 1 μM in a human liver microsome assay) and permeable to the BBB (log([brain concentration]/[blood concentration]) > −1). Solubility predictions at a neutral pH indicated moderate solubility (log S −6 to −4). The analysis suggests that both drug candidates have favorable ADME characteristics and potential as effective drugs, with 7MeERT showing stronger binding to NDUFA12. The strong predicted binding of 7MeERT to NDUFA12, along with favorable ADME profiles for both 7MeERT and Ertredin, highlights their potential as effective therapeutic agents.

### 3.4. CETSA of Ertredin and 7MeERT Binding to NDUFA12 and Their Effects on Thermal Stability

**Figure 4:**
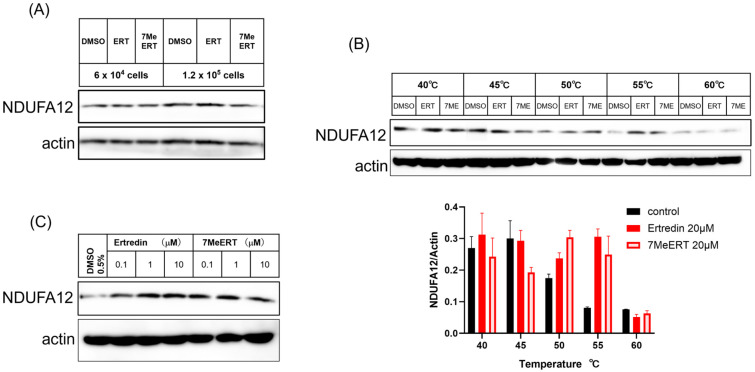
In EGFRvIII cells, the thermal stability of NDUFA12 is increased due to the addition of either Ertredin or 7MeERT. After treating the cells with 20 μM of Ertredin and 7MeERT for 1 h, they were subjected to thermal treatment using a thermal cycler at 40 °C (**A**), from 40 °C to 60 °C (**B**), and at 55°C (**C**) for 3 min. The cells were then cooled to 25 °C, solubilized, and analyzed by Western blot. The band intensity of NDUFA12 in (**B**) (**upper figure**) was quantified (**B**, **lower figure**). The full Western blot gel images with molecular weight markers for NDUFA12 in (**A**–**C**) are presented in Supplementary Figure S2, respectively.

Next, we performed a CETSA to investigate the effects of Ertredin and 7MeERT on thermal stability in U251/EGFRvIII cells and to confirm their binding to NDUFA12 (Figure 4). In U251/EGFRvIII cells at 6 × 10^4^ cells and 1.2 × 10^5^ cells, NDUFA12 remained stable at 40 °C regardless of the addition of Ertredin analogues (Figure 4A). On the other hand, thermal degradation of NDUFA12 was observed at temperatures above 50 °C, but it remained stable and showed thermal resistance when Ertredin or 7MeERT was added (Figure 4B). However, no thermal resistance was observed when heated to 60 °C after the addition of Ertredin analogues. The thermal resistance of NDUFA12 after the addition of Ertredin and 7MeERT at 55 °C was reaffirmed (Figure 4C), and the thermal stabilization of NDUFA12 by Ertredin in cells was consistent with previously generated results in HepG2 cells [8].

The CETSA results confirm that both Ertredin and 7MeERT stabilize NDUFA12 at elevated temperatures, with 7MeERT providing greater thermal stability. These findings support the hypothesis that both compounds bind to NDUFA12 and influence its thermal stability, consistent with earlier findings in HepG2 cells.

### 3.5. Impact of Ertredin and 7MeERT on Mitochondrial Energy Metabolism

**Figure 5:**
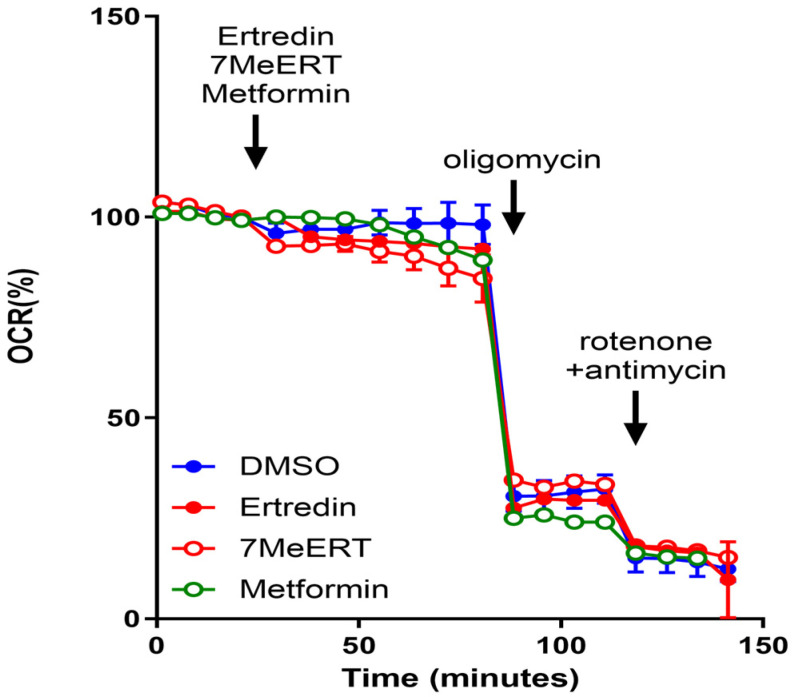
Ertredin and 7MeERT inhibit OCR in U251/EGFRvIII cells. OCR measurements were performed using a flux analyzer and an XF Mito Stress Test Kit after adding 0.1% DMSO (blue open circle), 50 μM Ertredin (red closed circle), 10 μM 7MeERT (red open circle), and 200 mM Metformin (green open circle) to U251/EGFRvIII cells.

Since NDUFA12 is a component protein of oxidative phosphorylation complex I, it is hypothesized that Ertredin and 7MeERT affect mitochondrial energy metabolism. Therefore, we used a Seahorse Flux Analyzer to measure the OCR (oxygen consumption rate) of cells after the addition of Ertredin and 7MeERT to examine their effects on oxidative phosphorylation. As shown in Figure 5, both Ertredin and 7MeERT exhibited OCR inhibitory activity, suggesting that they inhibit oxidative phosphorylation in U251/EGFRvIII cells. Metformin, which is known to inhibit complex I [20], also showed OCR inhibitory activity.

Both Ertredin and 7MeERT inhibit oxidative phosphorylation in U251/EGFRvIII cells by reducing the oxygen consumption rate (OCR). These findings suggest that the anticancer activity of these compounds is partly due to their inhibition of mitochondrial energy metabolism.

## 4. Discussion

The results show that 7MeERT, a methylated analogue of Ertredin, exhibits a higher antiproliferative activity than Ertredin in several cancer cell lines, including glioblastoma cells expressing EGFRvIII, epidermoid carcinoma A431 cells, cervical cancer HeLa cells, and multiple myeloma RPMI8226 cells. This suggests that the methylation of Ertredin enhances its antiproliferative effects.

The in silico docking analysis revealed that 7MeERT has a stronger binding affinity towards NDUFA12 compared to Ertredin, as indicated by the lower CDOCKER energy. The presence of the methyl group in 7MeERT significantly enhanced both π and alkyl interactions in the NDUFA12 binding, contributing to the increased binding energy that was observed. Despite the relatively weak nature of alkyl interactions in biology and chemistry, their cumulative effect on chemical reactivity could be significant [19].

The CETSA results confirmed that both Ertredin and 7MeERT bind to NDUFA12 and stabilize it at elevated temperatures. This thermal stabilization was consistent with previous findings in HepG2 cells [8]. The inhibition of oxidative phosphorylation by both compounds, as demonstrated by the Seahorse Flux Analyzer, further supports the hypothesis that they target NDUFA12, a component of oxidative phosphorylation complex I.

In addition, the present results show that similar to Ertredin, 7MeERT binds to NDUFA12 and exhibits significant antiproliferative activity. Previous studies have shown that 7MeERT is also a potent inhibitor of 3D spheroid formation in mouse cells, second only to Ertredin. Given the over 95% homology at the amino acid level between mouse and human NDUFA12, it is likely that this activity is mediated through interactions with NDUFA12. These results reinforce the potential role of 7MeERT in targeting mitochondrial functions, which is consistent with previous studies on Ertredin, and further suggest that 7MeERT may serve as a promising candidate for anticancer therapies.

Recently, Wu et al. compared the mRNA expression of the NDUFA12 gene in liver cell lines using the Illumina NovaSeq 6000 platform for RNA sequencing [21]. We also performed analyses using transcriptomic data from the GEO database (GEO Accession Number: GSE249370, GSE233708). The fragments per kilobase of transcript per million mapped reads (FPKM) values for NDUFA12 were analyzed in HL-7702 cells treated with 2 μM dimethyl sulfoxide and HepG2 cells treated with sg-control. These results indicated that NDUFA12 expression was approximately three times higher in the HepG2 liver cancer cell line compared to the normal liver cell line HL-7702 (Supplementary Figure S3), suggesting that NDUFA12 is overexpressed in liver cancer cells and may be a promising biological target for liver cancer treatment.

Collectively, these results suggest that both 7MeERT and Ertredin inhibit energy metabolism and proliferation in various human cancer cells by targeting NDUFA12. Notably, 7MeERT exhibited a higher antiproliferative activity compared to Ertredin, making it a promising candidate for further development as an anticancer agent.

Given the interesting results of this study, a comprehensive analysis of the specificity of 7MeERT for NDUFA12 compared to other potential targets will be required for further investigations. While molecular docking and CETSA data strongly suggest that NDUFA12 is a primary target, off-target effects cannot be completely excluded. Future studies using proteomic approaches could help identify any additional binding partners and further clarify the selectivity of 7MeERT across a broader range of mitochondrial and non-mitochondrial proteins. In addition, further studies in animal models of glioblastoma and other cancers are needed to better evaluate the therapeutic potential of 7MeERT.

Finally, the exploration of combination therapies combining 7MeERT with other metabolic inhibitors or immune checkpoint inhibitors may yield synergistic effects that enhance its anticancer properties. Future studies on the pharmacokinetics and pharmacodynamics of 7MeERT in different cancer types will be critical to determine its therapeutic window and inform dosing strategies in preparation for clinical trials.

## 5. Conclusions

This study demonstrated that 7MeERT, a methylated analogue of Ertredin, exhibits enhanced antiproliferative activity in several cancer cell lines compared to Ertredin. Our results suggest that the methyl group in 7MeERT plays a critical role in its increased binding affinity to NDUFA12, as indicated by the molecular docking analysis. Furthermore, the CETSA results showed that both compounds stabilize NDUFA12 and influence its thermal stability, while the OCR measurements confirmed that 7MeERT and Ertredin inhibit oxidative phosphorylation in U251/EGFRvIII cells. These results support the hypothesis that NDUFA12 is a viable therapeutic target for mitochondrial-driven cancers, and that the higher binding affinity of 7MeERT may make it a more effective anticancer agent. Exploring the role of NDUFA12 in other cancer types and understanding how mitochondrial metabolism contributes to cancer progression will be important areas for future research.

## Figures and Tables

**Table 1:** Growth inhibition of various cells by Ertredin and 7MeERT.

CELLS		IC50 (μM)	
		Ertredin	7MeERT
3D	NIH3T3/EGFRvIII	0.04	0.1
2D	NIH3T3	INACTIVE	INACTIVE
	U251/EGFRvIII	10	2
	U251/vector	5	2
	U87MG/EGFRvIII	20	2
	U87MG/vector	20	5
	A431	50	5
	HELA	50	1
	RPMI8226	16	8
	KMS11	>50	>50

**Table 2:** Physicochemical Properties (ADME/T) Prediction.

Hyper ADME/T	7MeERT	Ertredin
Structure	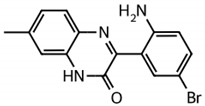	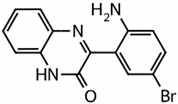
Chemical formula	C15H12BrN3O	C14H10BrN3O
Molecular weight	329	315
Binding score with NDUFA12	−3.7 kcal/mol	−3.3 kcal/mol
TPSA (A^2^)	72	72
Lipinski rule	Pass	Pass
Veber’s (GSK) rule	Pass	Pass
Log P	3.2	2.9
Solubility	Moderate	Moderate
Metabolic stability	Stable	Stable
Druglikeness	71.5	82.6
BBB	Permeable	Permeable

## Data Availability

Data are contained within the article.

## Supplementary Materials

The following supporting information can be downloaded at: https://www.mdpi.com/article/10.3390/biom14091197/s1, Figure S1: In glioblastoma cells transduced with the EGFRvIII gene, EGFRvIII protein expression was observed; Figure S2: Full Western blot gel images with molecular weight markers for NDUFA12; Figure S3: NDUFA12 RNA-seq expression level.

## Abbreviations

EGFR	Epidermal Growth Factor Receptor
EGFRvIII	Epidermal Growth Factor Receptor variant III
NDUFA12	NADH Dehydrogenase (Ubiquinone) 1 Alpha Subcomplex Subunit 12
3D	Three-Dimensional
2D	Two-Dimensional
CETSA	Cellular Thermal Shift Assay
OCR	Oxygen Consumption Rate
DMSO	Dimethyl Sulfoxide
PBS	Phosphate-Buffered Saline
FBS	Fetal Bovine Serum
SDS-PAGE	Sodium Dodecyl Sulfate Polyacrylamide Gel Electrophoresis
MTT	3-(4,5-Dimethylthiazol-2-yl)-2,5-Diphenyltetrazolium Bromide
RPMI	Roswell Park Memorial Institute
IC_50_	Half Maximal Inhibitory Concentration
TPSA	Topological Polar Surface Area
ADME/T	Absorption, Distribution, Metabolism, Excretion, and Toxicity
logP	Partition Coefficient between Water and Octanol
