# Supplementary material for: Enhanced Anticancer Activity of 7MeERT over Ertredin: A Comparative Study on Cancer Cell Proliferation and NDUFA12 Binding
Source: Biomolecules. 2024 Sep 23;14(9):1197. doi: 10.3390/biom14091197 (PMC11430042; doi:10.3390/biom14091197)

Supplementary information:

Materials:

For the Supplementary Western blotting, rabbit anti-EGFR antibody and rabbit anti-EGFR P-Tyr 1068 antibody were purchased from Cell Signaling Technology, and mouse anti-GAPDH antibody was purchased from Santa Cruz.

Figure legends

Supplementary Figure S1:

In glioblastoma cells transduced with the EGFRvIII gene, EGFRvIII protein expression was observed. U251/EGFRvIII or U251/vector (A) cells ($5x10^5$ cells) and U87MG/EGFRvIII or U87MG/vector (B) cells ($6x10^5$ cells) were cultured for 24 hours after seeding. Following this, Ertredin at various concentrations was added and incubated for an additional 24 hours. Whole cell lysates were then prepared, and 15 micrograms of protein from each lysate was applied to SDS-PAGE for Western blot analysis.

Supplementary Figure S２: Full Western blot gel images with molecular weight markers for NDUFA12. (A) shows the Western blot after thermal treatment at 40°C for 3 minutes. (B) shows the Western blot after thermal treatment from 40°C to 60°C. (C) shows the Western blot after thermal treatment at 55°C for 3 minutes. Molecular weight markers are indicated on the right side of each image.

Supplementary Figure S３: NDUFA12 RNA-seq expression level.

Supplementary Figure S1

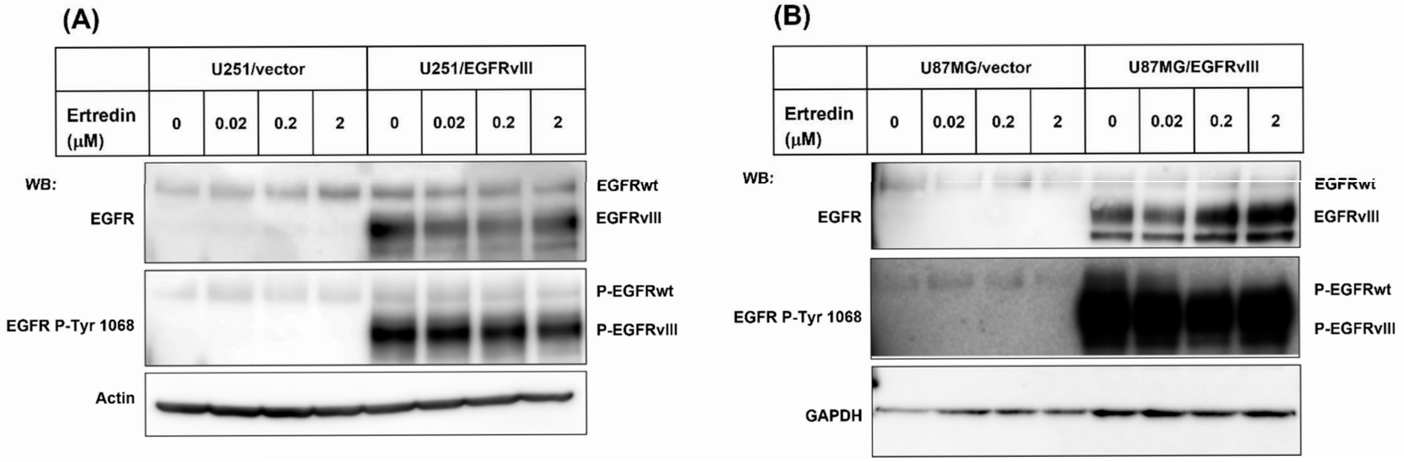

Supplementary Figure S２:

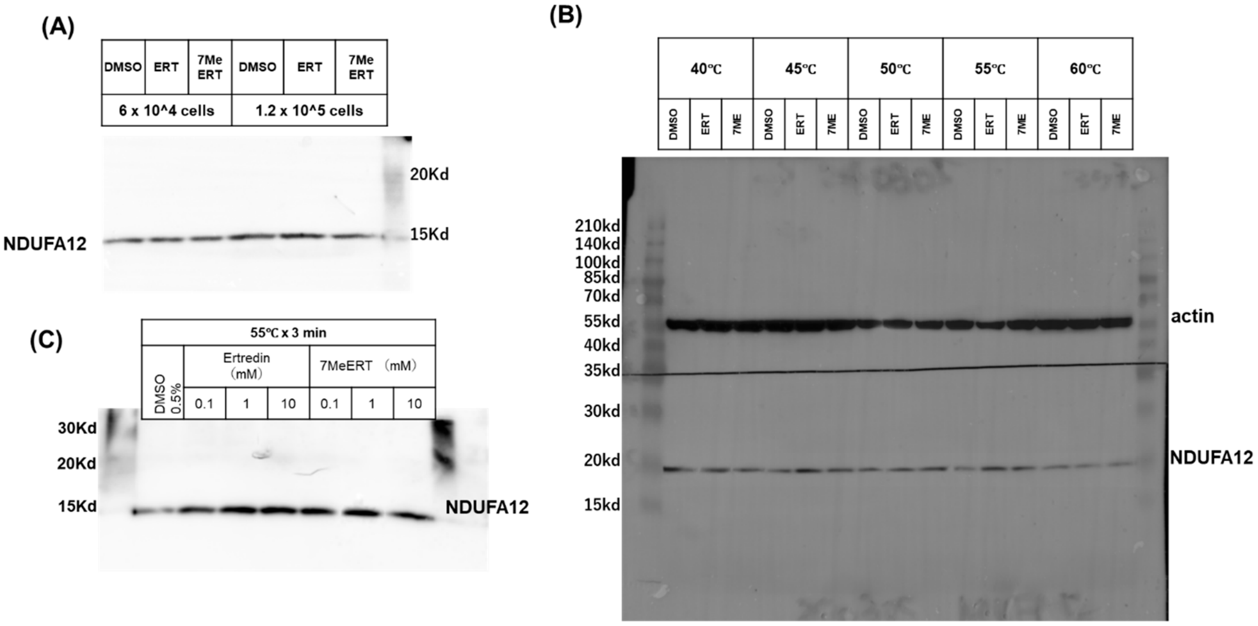

Supplementary Figure S３:

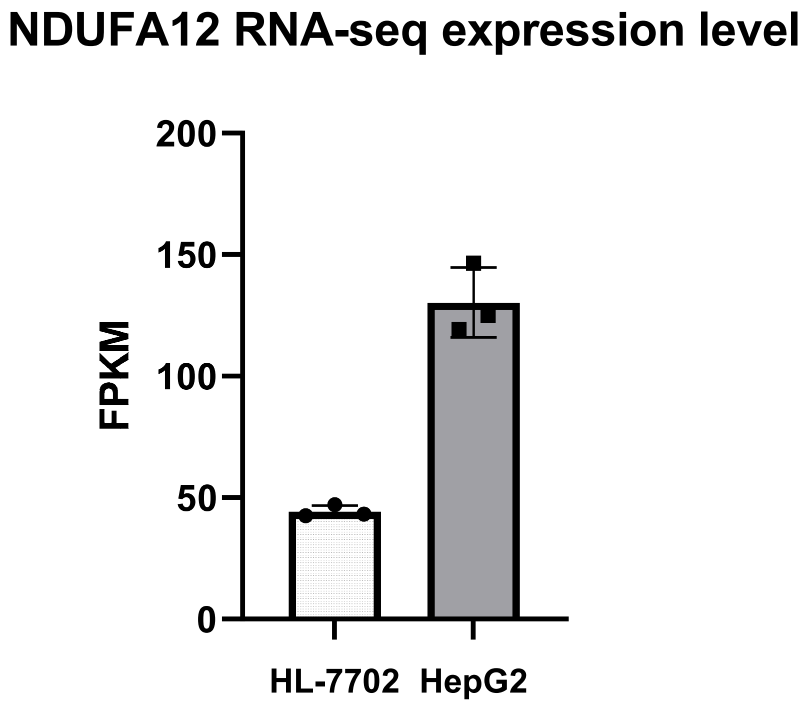

Supplement: Supplementary file 1 [file biomolecules-14-01197-s001.zip › biomolecules-3170141-supplementary.pdf]
